# Molecular structure analysis and biological properties investigation on antiseptic drug; 2-amino-1-phenyl-1-propanol using spectroscopic and computational research analysis

**DOI:** 10.1016/j.heliyon.2021.e06699

**Published:** 2021-04-07

**Authors:** A. Abbas Manthiri, S. Ramalingam, Gene George, R. Aarthi

**Affiliations:** a Department of Physics, Jamal Mohamed College, Tiruchirappalli, Tamilnadu, India; b Department of Physics, A.V.C. College, Mayiladuthurai, Tamilnadu, India; c Department of Physics, T.B.M.L. College, Porayar, Tamilnadu, India; d Department of Physics, ST. Theresa's Arts and Science College, Tharangambadi, Tamilnadu, India

**Keywords:** 2-Amino-1-phenyl-1-propanol, FT-IR, FT-Raman, NMR, Structure activity, Biological profile, Biological ambiance, Chemical property, Enantiomer

## Abstract

The inducement of physical, chemical, structural and biological properties to entice of pharmaceutical property was analyzed by Vibrational spectroscopic, biological and theoretical tools. The structural arrangement for describing structure activity was investigated by injecting ligand groups in internal coordinate system by molecular tools (FT adopted IR, Raman, and NMR). Bond length and bond angle strain was pronounced much due to the chemical equivalent forces extension due to the injection of substitutional groups on base compound and thus non-Centro symmetry was processed. The molecular charge depletion profile was thoroughly studied to persuade protonic and electronic delocalization setup for arranging the drug potential. The chemi-equivalent potential exchange was monitored among different parts of the molecule for obtaining drug mechanism. The biological profile was keenly observed to look at the biological ambiance of the present molecule to fabricate advanced drug. The Lipinski five rule parameters; M_V_ = 137.18, LogP = 0.27, HBD = 2, HBA = 2 and TPSA = 46.2 A^2^ showed the enhancement of additive drug quality. The exchange of oscillating chemical energy in the core and allied carbons of the base skeleton was keenly noted to find the prearranged chemical environment for successful drug mechanism. The non bonded transitions between Lewis acid and base of bonded molecular system were observed to determine the restoring potential to customize drug potential. The drug assistance for enantiomer characteristics of chirality sequence was displayed to expose the toxicity effect of the molecule. The active molecular bondings on different sites of molecule were measured by estimating polarizability and associated biological inhibition was validated.

## 1 Introduction

2-Amino-1-phenyl-1-propanol is chiral-active amino derivative which belongs to psychoactive drug family and the application of such base compound was to relieve nasal congestion and also acted as anorectic agent [1]. This specific chemical compound is fabricated in the base of Phenyl ring in which the hydrocarbon group (CH_2_CH_3_) is injected jointly with aliphatic substitution along with amino group. Such amino group stressed the joining chain very much and thereby the proportionate chain strain was observed on the alternation of bond parameters. According the literatures [2, 3, 4], the compound; 1-phenyl-1-propanol under study, is anti-depression drug and the fundamental pharmaceutical function is altered and compound become psychoactive drug due to the operative electronegative amino group [5, 6]. As per the literature [7], present com pound has antifungal potency which was stimulated by the injection of amino group in ethyl part. By the application of biological reaction mechanism, the present molecule possessed bioactivity [8] and also acted as catalytic ability.

**Figure 1:**
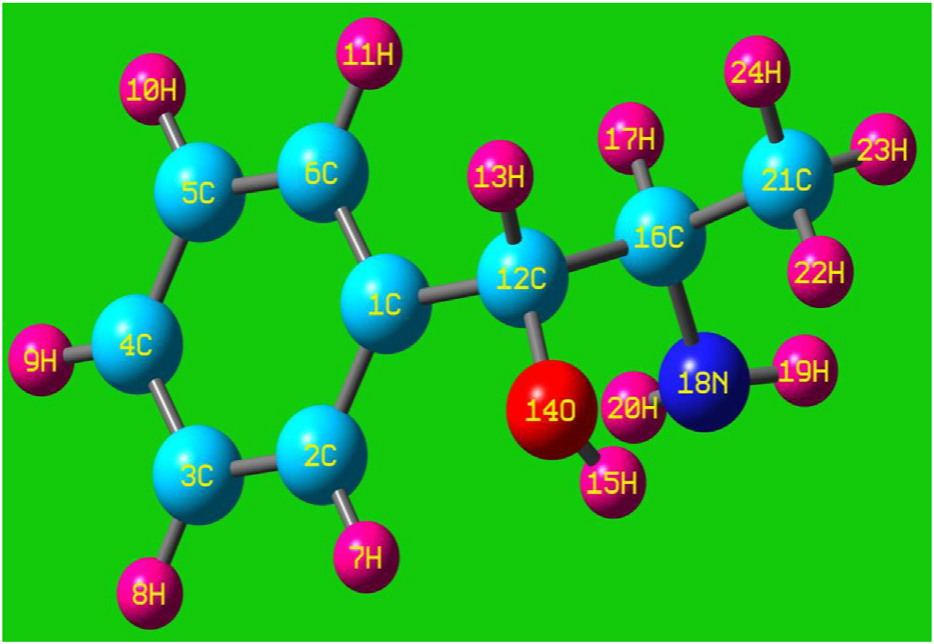
Model structure of 2-Amino-1-phenyl-1-propanol.

Usually the pharmacophore fragment combination; hydroxyl-amino group (pharmaceutical active moiety) is inducing bioactive potential in the chemical species and in this case, such molecular amalgamation is purposely customized to monitor the drug activity. The *Cis-Trans* structure complexion formation of present molecular complex ratify specific drug potent along with optimized structure. Here the propanol content forcefully possesses a suitable drug agent by the stimulant of the amino group and thereby acts as a norepinephrine releasing drug. It is also called phenyl propanol amine which is usually found in the biological fluids and is used for pharmaceutical formulation [9, 10]. Though the present compound is obsessed psychoactive drug activity and possessed wide drug importance, no recent work found on the prediction of unknown properties of 2-Amino-1-phenyl-1-propanol. In this attempt of research work, the molecular structural, pharmaco-biological, rot-vibrational and unknown physico-chemical properties were predicted to use this compound for further drug advancement. The entire analyses have been made on the model structure as shown in Figure 1 where it was ensured that, the structure was free from *trans* and *cis* from.

## 2 Experimental details

The organic chemical species; 2-Amino-1-phenyl-1-propanol is was purchased by UKAVA Chemical industry as raw material, USA and it was further isolated and filtered, thus it was trusted to be advanced spectroscopic grade and generally it is preferred to record the spectra. The FT-IR frequency prototype for all fingerprint region and group frequency region was obtained with the application of IR-Bruker IFS; 171V equipped with high intensity-depth scanning speed [11]. The FT-Raman wavenumber pattern was also registered from the same instrument by making several scanning processes to avoid unwanted summed peaks which facilitated with Raman module. High resolution^1^H NMR and ^13^C NMR spectral pattern were mapped using 600 MHz and 125 MHz FT-NMR spectroscope with high magnetic slope [12]. The NMR spectra were resolved using TDS processes in order to make coherent with calculated spectra. The UV-Vis absorption peaks were determined at solid phase in the region of 50 nm–700 nm, with the scanning rate of 0.50 nm, using the UV-1900 version instrument. The absorption peaks were verified with vibrational region wavenumbers and consecutively the absorption peak shift was tested.

## 3 Computational methods

The computational calculations were performed using hybrid methodological, such as Lee-Yong-Per theory and to compute structural and optimized parameters and validated by drafting allied chemical calculations on latest Gaussian 16 series software in i MAC 4C system [13]. The mulliken charge level and vibrational frequency pattern was calculated on hybrid computations such as B3LYP/6–311++G(d,p). The isotropic and anisotropic chemical shift was computed to opt the NMR-GIAO method by the I-PCM model as a hybrid calculation method. The entire calculations were performed for calculating biological property using HyperChem tool on 8.0 versions and were evaluated by literature values. The molecular electrostatic potential regions are determined and depicted in the figure. The multipole moment, hypo-polarizability and hyper-polarizability in different internal coordinates were calculated by the same hybrid methods [14]. The helical characteristics VCD model peak pattern was recorded and the enantiomer property of the compound was understood to reveal toxicity effects.

## 4 Discussions on results

### 4.1 Structural importance analysis

**Figure 2:**
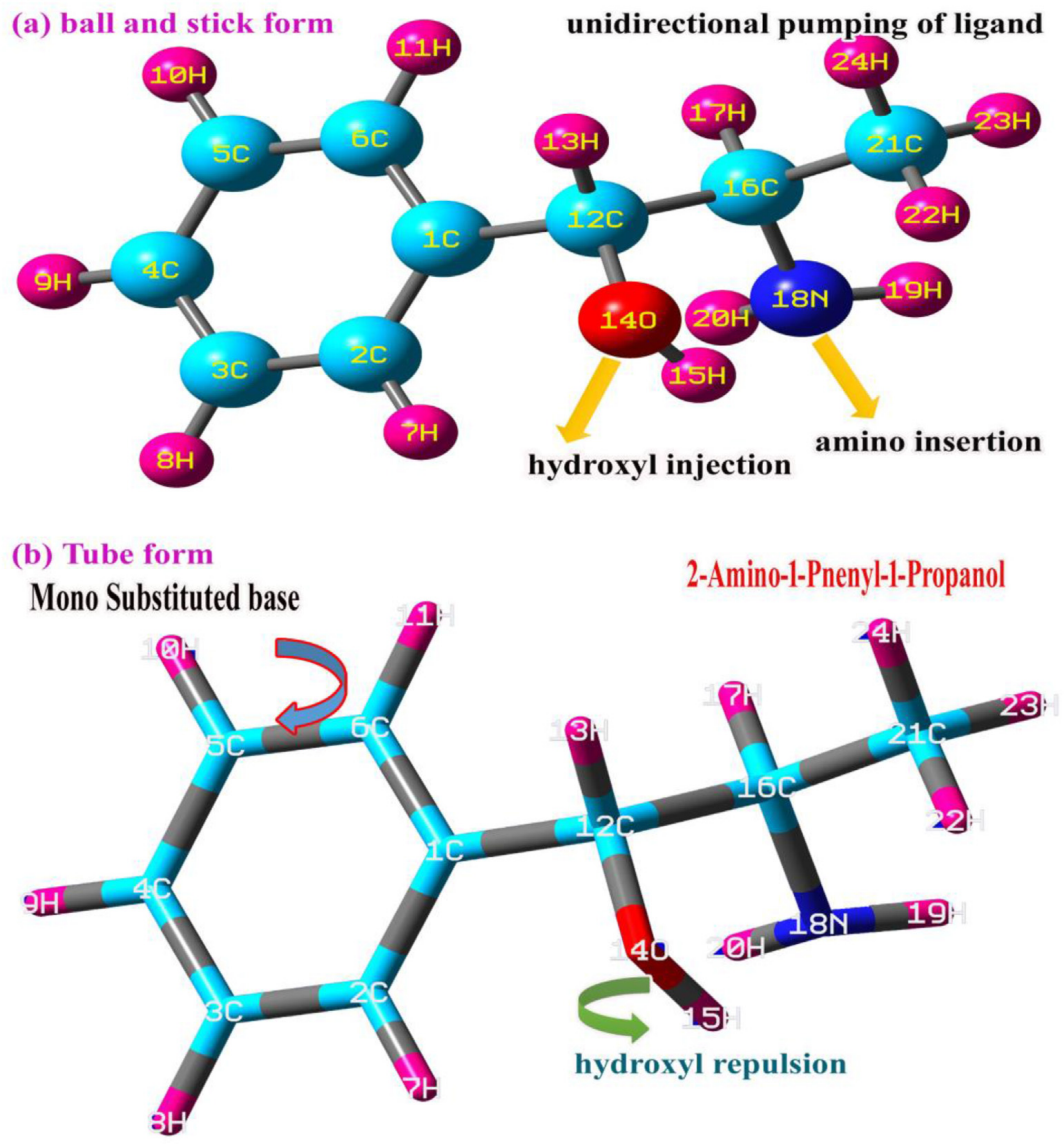
Model Structure: (a) Ball and stick form (b) Tube form of 2-Amino-1-phenyl-1-propanol.

As for as optimization process of structure is concern, regularly molecular structure is optimized after the arrangement of bond length and bond angle by intermolecular attractive and repulsive forces exist among molecular site. The negative and positive charge domain is displaced and oriented in order to equalize chemical equilibrium forces with respect to passive and active ligand group injection over base compound. The displacement of charge levels at the centre of symmetry of base frame is depending upon unidirectional and multidirectional insertion of ligand groups. The entire alternation of bond parameters describes the impact of ligand group over the base framework and thereby the physical and chemical properties are modified. All the bond length and bond angles in alternated condition by fully mass loaded ligands are shown in Figure 2 and distorted bond lengths are portrayed in Table 1.

**Table 1:** Optimized geometrical parameters for 2-Amino-1-phenyl-1-propanol.


Geometrical Parameters	Methods				
	HF	B3LYP		B3PW91	
	6-311++G (d, p)	6-31++G (d, p)	6-311++G (d, p)	6-31++G (d, p)	6-311++G (d, p)
Bond length (Å)					
C1–C2	1.387	1.400	1.396	1.397	1.394
C1–C6	1.393	1.404	1.401	1.402	1.399
C1–C12	1.526	1.529	1.528	1.524	1.523
C2–C3	1.389	1.399	1.396	1.397	1.394
C2–H7	1.073	1.085	1.083	1.086	1.084
C3–C4	1.383	1.396	1.393	1.394	1.390
C3–H8	1.076	1.087	1.085	1.087	1.086
C4–C5	1.387	1.399	1.395	1.397	1.393
C4–H9	1.076	1.086	1.084	1.087	1.085
C5–C6	1.383	1.396	1.392	1.394	1.390
C5–H10	1.076	1.087	1.085	1.087	1.086
C6–H11	1.077	1.088	1.086	1.089	1.087
C12–H13	1.085	1.098	1.095	1.098	1.097
C12–O14	1.396	1.418	1.417	1.410	1.409
C12–C16	1.543	1.558	1.556	1.553	1.550
O14–H15	0.945	0.976	0.972	0.976	0.972
C16–C17	1.089	1.100	1.098	1.101	1.099
C16–N18	1.461	1.474	1.473	1.467	1.467
C16–C21	1.526	1.530	1.528	1.525	1.523
N18–H19	0.999	1.015	1.014	1.014	1.013
N18–H20	1.000	1.017	1.015	1.016	1.015
C21–H22	1.084	1.094	1.092	1.094	1.093
C21–H23	1.087	1.096	1.095	1.096	1.095
C21–H24	1.086	1.095	1.093	1.094	1.093
Bond angle (°)					
C2–C1–C6	118.41	118.57	118.58	118.62	118.63
C2–C1–C12	122.00	121.67	121.67	121.46	121.50
C6–C1–C12	119.59	119.76	119.75	119.92	119.88
C1–C2–C3	120.66	120.63	120.62	120.62	120.60
C1–C2–H7	119.67	119.04	119.12	118.88	118.95
C3–C2–H7	119.66	120.31	120.25	120.49	120.43
C2–C3–C4	120.45	120.36	120.36	120.34	120.35
C2–C3–H8	119.53	119.62	119.64	119.64	119.65
C4–C3–H8	120.01	120.02	120.00	120.02	120.00
C3–C4–C5	119.33	119.45	119.46	119.46	119.47
C3–C4–H9	120.38	120.33	120.33	120.32	120.32
C5–C4–H9	120.28	120.22	120.21	120.21	120.20
C4–C5–C6	120.08	120.05	120.06	120.06	120.06
C4–C5–H10	120.11	120.13	120.10	120.11	120.09
C6–C5–H10	119.80	119.82	119.85	119.83	119.85
C1–C6–C5	121.05	120.93	120.92	120.89	120.88
C1–C6–H11	119.81	119.75	119.77	119.76	119.76
C5–C6–H11	119.14	119.32	119.31	119.35	119.36
C1–C12–H13	107.53	108.07	107.99	108.13	108.06
C1–C12–O14	112.40	112.72	112.79	112.76	112.86
C1–C12–C16	112.03	111.72	111.67	111.37	111.26
H13–C12–O14	106.45	106.74	106.65	107.17	107.08
C13–C12–C16	108.01	108.00	108.08	108.17	108.24
O14–C12–C16	110.14	109.35	109.41	109.05	109.15
C12–O14–H15	107.81	105.12	105.16	104.36	104.40
C12–C16–H17	107.80	107.94	107.92	108.13	108.08
C12–C16–N18	108.03	106.84	106.90	106.41	106.49
C12–C16–C21	111.57	111.79	111.72	111.66	111.62
H17–C16–N18	111.75	112.22	112.13	112.39	112.30
H17–C16–C21	108.15	108.59	108.65	108.75	108.82
N18–C16–C21	109.56	109.49	109.53	109.52	109.56
C16–N18–H19	111.59	111.54	111.46	111.58	111.46
H16–N18–H20	111.63	111.42	111.39	111.29	111.22
H19–N18–H20	107.83	107.93	107.91	108.00	107.95
C16–C21–H22	111.24	110.99	110.99	110.87	110.87
C16–C21–H23	110.46	110.77	110.75	110.89	110.86
C16–C21–H24	110.60	110.54	110.58	110.60	110.62
H22–C21–H23	108.38	108.50	108.47	108.49	108.46
H22–C21–H24	108.34	108.25	108.26	108.23	108.24
H23–C21–H24	107.72	107.68	107.68	107.65	107.67
Dihedral angle (°)					
C6–C1–C2–C3	0.91	0.90	0.89	0.90	0.89
C6–C1–C2–H7	177.94	177.72	177.73	177.73	177.74
C12–C1–C2–C3	-179.33	-179.08	179.03	-178.94	-178.91
C12–C1–C2–H7	1.82	2.30	2.36	2.43	2.47
C2–C1–C6–C5	-0.79	-0.81	-0.77	-0.80	-0.76
C2–C1–C6–H11	178.52	178.51	178.50	178.51	178.49
C12–C1–C6–C5	179.44	179.17	179.15	179.05	179.04
C12–C1–C6–H11	-1.25	-1.51	-1.58	-1.65	-1.71
C1–C2–C12–H13	-130.55	-130.34	130.37	-129.64	-129.68
C1–C2–C12–O14	-13.72	-12.62	-12.77	-11.33	-11.47
C2–C1–C12–C16	110.92	110.98	110.93	111.64	111.62
C6–C1–C2–H13	49.21	49.68	49.72	50.52	50.53
C6–C1–C12–O14	166.04	167.40	167.32	168.83	168.74
C6–C1–C12–C16	-69.32	-69.00	-68.98	-68.20	-68.17
C1–C2–C3–C4	-0.38	-0.34	-0.35	-0.35	-0.35
C1–C2–C3–H8	-179.92	-179.87	179.88	-179.87	-179.88
H7–C2–C3–C4	178.47	178.25	178.25	178.25	178.26
H7–C2–C3–H8	-1.07	-1.28	-1.28	-1.27	-1.27
C2–C3–C4–C5	-0.29	-0.31	-0.32	-0.32	-0.34
C2–C3–C4–H9	-179.72	-179.74	179.73	-179.75	-179.73
H8–C3–C4–C5	179.25	179.22	179.20	179.20	179.19
H8–C3–C4–H9	-0.18	-0.21	-0.20	-0.23	-0.21
C3–C4–C5–C6	0.41	0.39	0.44	0.42	0.47
C3–C4–C5–H10	-179.18	-179.16	179.13	-179.16	-179.12
H9–C4–C5–C6	179.84	179.82	179.85	179.85	179.86
H9–C4–C5–H10	0.26	0.27	0.28	0.28	0.28
C4–C5–C6–C1	0.14	0.18	0.11	0.15	0.09
C4–C5–C6–H11	-179.18	-179.16	-179.17	-179.16	-179.16
H10–C5–C6–C1	179.72	179.73	179.68	179.72	179.68
H10–C5–C6–H11	0.41	0.40	0.40	0.41	0.42
C1–C1–O14–H15	88.75	91.71	90.91	91.91	91.01
H13–C11–O14–H15	-153.77	-149.79	150.70	-149.22	-150.21
C6–C12–O14–H15	-36.92	-33.20	-34.02	-32.35	-33.26
C1–C12–C16–H17	50.49	46.77	47.06	46.32	46.79
C1–C12–C16–N18	-70.43	-74.11	-73.74	-74.63	-74.06
C1–C12–C16–C21	169.09	166.13	166.44	165.92	166.42
H13–C12–C16–H17	-67.75	-71.95	-71.59	-72.37	-71.79
H13–C12–C16–N18	171.33	167.17	167.61	166.68	167.36
H13–C12–C16–C21	50.84	47.41	47.79	47.23	47.83
O14–C12–C16–H17	176.37	172.26	172.65	171.39	171.99
O14–C12–C16–N18	55.46	51.38	51.85	50.45	51.14
O14–C12–C16–C21	-65.03	-68.38	-67.97	-69.01	-68.38
C12–C16–N18–H19	-167.01	-165.68	165.26	-165.31	-165.26
C12–C16–N18–H20	72.25	73.64	74.15	73.98	74.22
H17–C16–N18–H19	74.57	76.21	76.65	76.52	76.63
H17–C16–N18–H20	-46.17	-44.48	-43.94	-44.20	-43.89

In the present case, the mono substitution was observed at *ortho* position of benzene ring and ligand chemical impact is pronounced in unidirectional manner. Here, due to the ligand chain, the chemical potential gradient of benzene ring was altered certainly and that chemical property to be on par with the ligand group. In the substitutional process, there was charge repulsion taking place between negative and positive content of OH and NH_2_. So, the chain was not linear and the ligand placed in different planes and the magnitude of dipole moment is dispersed in different dimensions. The bond length of core CC was found to be deferred due to the substitution chain. Particularly, the bond length C1–C2 and C1–C6 were determined more stretched than rest of other CC due to the substitutions. Here, the reception of ligand group was well noticeable from the bond length elongation. Though bond length was stretched at the place of substitution, the entire core bonds are found to be rather affected. Apart from the benzene ring, in the ligand chain, the repulsive forces were observed between CC of the substitutional sequence. Particularly, the bond length abruptly elongated to be 0.135 and 0.163Å in the place of C1–C12, C12–C16 and C16–C21 respectively. In addition to that, due to the repulsive forces exerted between the same charge gradient domains, the bond angle C12–O14–H15 was moved away from the linear position than C16–N18–H19. These two bond angles and dihedral angle twisted in opposite internal coordinates. Because of this twisting of angle in the chain, the centre of symmetry in the molecule was shifted towards the ligand group which affects non-Centro symmetry of the molecular system. This leads hyper active polarization inside the molecular site and thereby the biological contribution of the molecule is improved.

### 4.2 Molecular charge dispersion sketch

**Figure 3:**
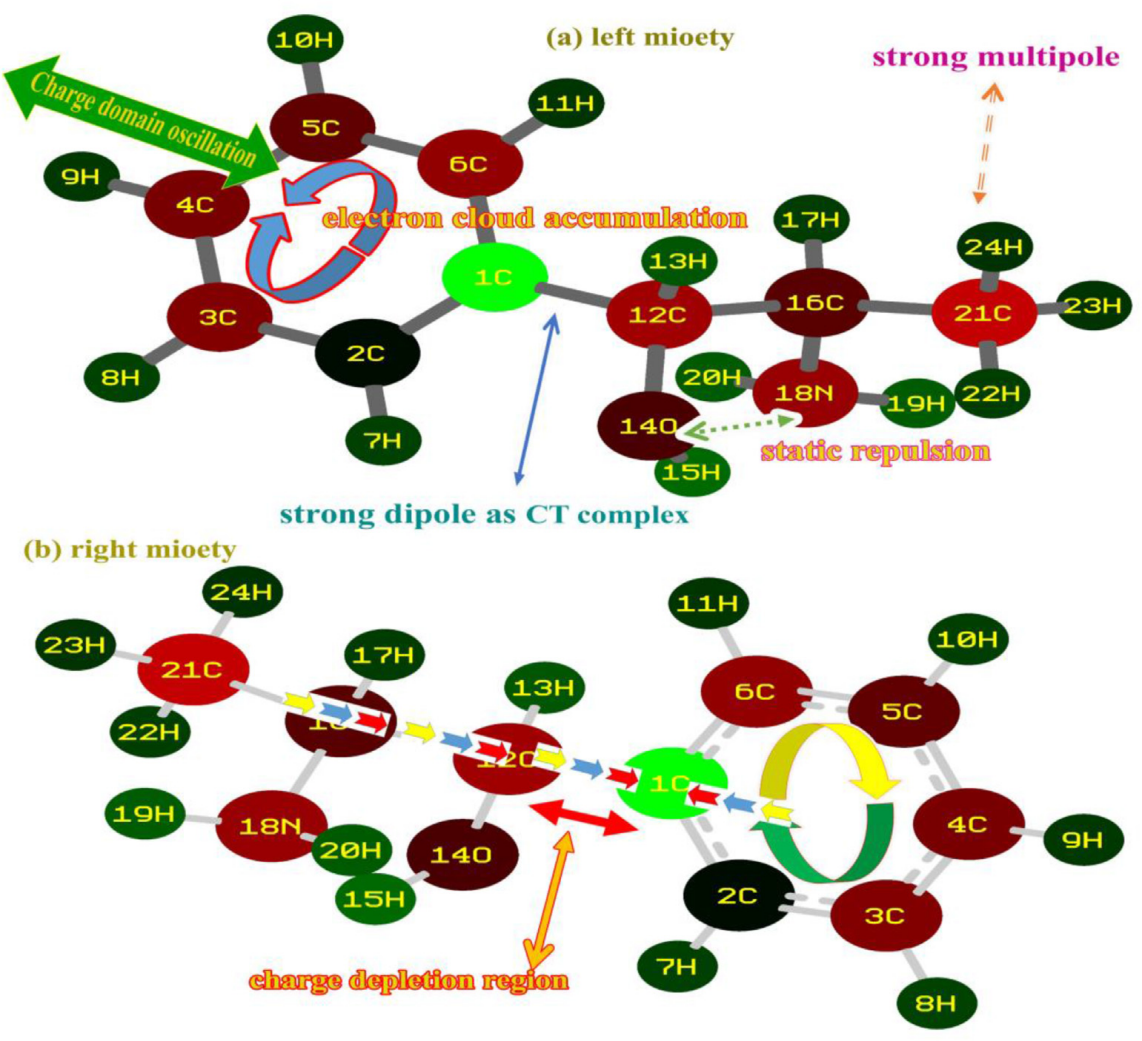
(a) Left moiety (b) Right moiety mulliken charge level of 2-Amino-1-phenyl-1-propanol.

Usually, the molecular charge levels are concentrated over the core carbons of the benzene ring when it is not substituted. Simultaneously, the concrete physical and chemical properties are sustained over benzene skeleton. But, if hexagonal ring is substituted, the molecular charge levels would be reoriented on the chemical equilibrium forces which are guided by the charge influence of ligand groups. So that, the entire chemical as well structural properties are altered regarding the mass and charge level enticement of injection of ligands. The mulliken charge levels (molecular charge assignment) dispersion unswervingly or circuitously illustrates the charge assignment over entities which forcefully drive the chemical environment in different places in chemical species. The molecular charge dispersion view for the present compound is shown in Figure 3.

In this case, mono substitution is possible due to the injection of chain and erratically the electron cloud was disseminated in the resulting molecule. The injection of ligands was made on C1 of the ring due to which the entire CC of the ring was suddenly altered in the form of negative population such that, except C1 and C2, the electron cloud was populated on the entire core CC. This was found to be unstable due to the oscillation of charges pumped by the ligand groups which was evident by all the nodal atoms C, O and N were appeared to be negatively (looks like red atom) charged as shown in Figure 2. The centre of symmetry of terminal charge domain zone C1, showed the main oscillation centre in which the charge domain was found to be oscillated between ring and chain. The formation of strong dipoles in ligand and hexagonal rings appeared over C1–C12, CH of methyl group and NH of amino group which caused the endothermic existence of active chemical potential for inducing antibiotic and psychotic activity. The charge depletion path in the molecule was clearly shown in the figure to understand purposive charge displacement. The mulliken charge dispersive process itself provides clear evidence for the generation of CT complexes and it was surprisingly identified as a C–C bridge bond.

### 4.3 Biological studies

**Figure 4:**
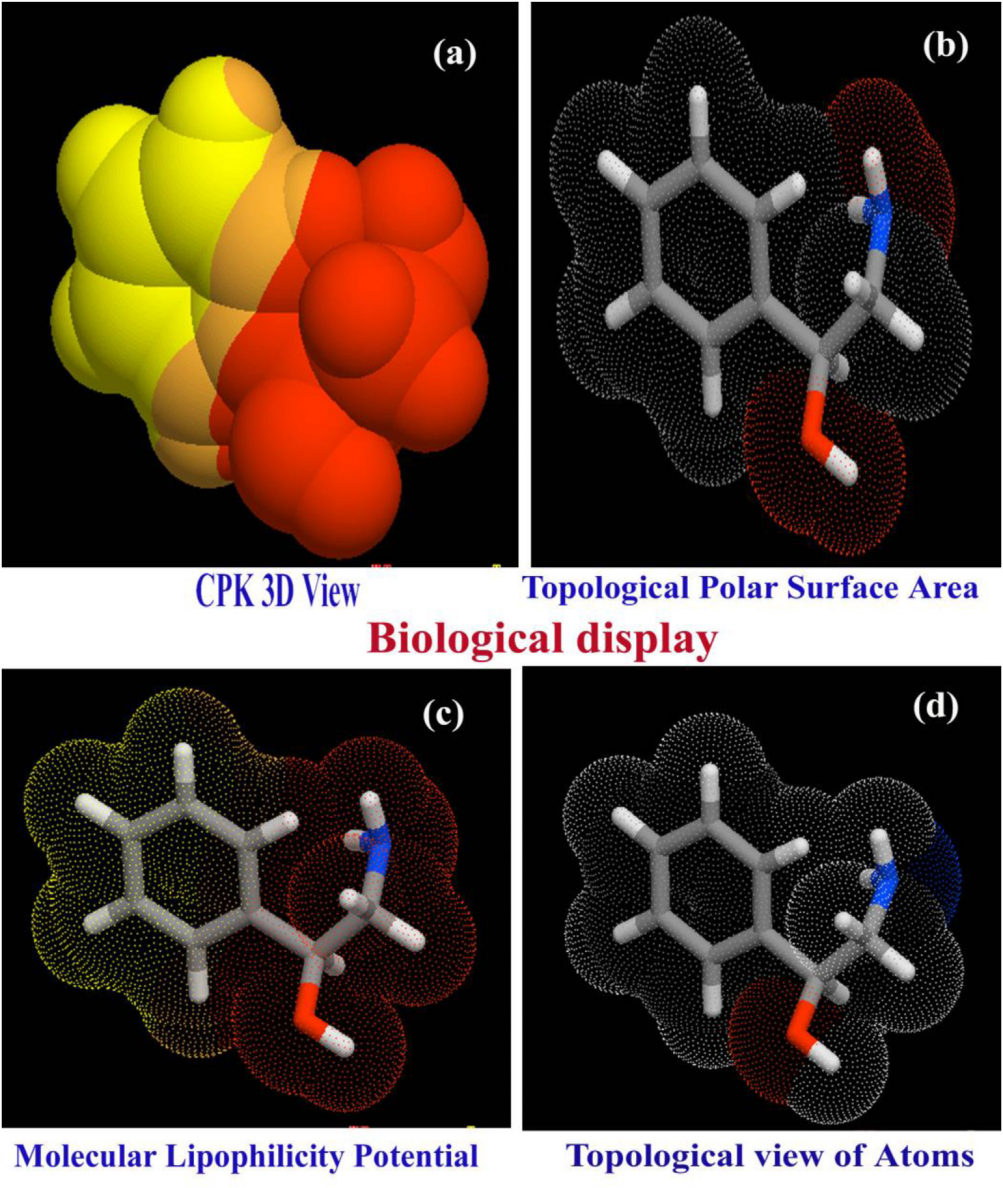
(a) CPK view (b) TPSA(c) MLP (d) Topological view of 2-Amino-1-phenyl-1-propanol.

The conception of drug-likeness provides useful information for handling the chemical agents for the drug formulation for preparing the successful drug. The Drug-likeness is evaluated the physicochemical properties related to produce the impact in terms of molecular behavior in vivo, with meticulous respect to molecular solubility, permeability, metabolic steadiness and carrier effects [15]. Certainly, the drug-likeness is frequently used as a substitute for oral bioavailability [16]. The biological parameters for optimized drug molecules were computed by applying required structural information on Molinspiration program and evaluated variables for the same are obtained in Table 2. The respective lipophilicity profile is demonstrated in Figure 4.

**Table 2:** Calculated biological parameters of 2-Amino-1-phenyl-1-propanol.

Parameters	values
Hydrogen bond donor count	2
Hydrogen bond acceptor count	2
Rotatable bond count	2
Topological Polar Surface Area	46.2 A^2^
Mono isotopic Mass	151.1 g/mol
Heavy Atom Count	11
Covalently-Bonded Unit Count	1
Log p	0.27
n atoms	10
MW	137.18
n ON	2
n OHNH	3
n violations	0
n rotb	2
volume	136.98
GPCR ligand	-0.35
Ion channel modulator	-0.23
Kinase inhibitor	-0.75
Nuclear receptor ligand	-1.12
Protease inhibitor	-0.77
Enzyme inhibitor	-0.15

The most common assessment of drug-likeness of the organic and inorganic species is obviously the rules and familiarly called Lipinski's Rule of Five that states that chemical compound is more likely to show evidence of privileged absorption or permeation when five physicochemical criteria are fulfilled [17]. Such that, the chemical compound used for drug formulation should have the molecular mass not to be greater than 500, the measured octanol-water partition coefficient such as log P, is to be less than five, less than five HBD, the number of hydrogen-bond acceptors not to be greater than ten and the Topological Polar Surface Area is to be less than 140 A^2^. The present molecule was having M_V_ = 137.18, LogP = 0.27, HBD = 2, HBA = 2 and TPSA = 46.2 A^2^ in that order. All the parametric values were found to be very low and the present compound is able to have additional substitutional groups for the enhancement of additive drug quality. Even the compound possessed very large substitutional groups, its quantity of Lipinski parametric rate will not be crossed the limit.

Heavy atom count and rotatable bond count is generally affecting the optimization and toxicity rate of the chemical species and present case have 11 and 2 respectively for the same. The title molecule does not have *trans* and *cis* structural formation naturally and heavy atom was not necessarily replaced. Simultaneously, since the heavy atom was limited up to O and N, present chemical species were not in critical toxic condition. The GPCR ligand of this case was detected to be 0.35, was moderate to pass through the transmembrane system and actively penetrate in to appropriate protein complexes. The ion channel modulating ability value was measured as 0.23 which was very low for this case and could be interacted with protein directly and guiding to change the action potentials and able to have penetration capability for electrical signals across the membrane [18]. The Kinase inhibitor was assessed to be 0.75 which is less than unity and was found to be moderate to inhibit the continuous action of the process directly controlling the activity. Since the Nuclear receptor ligand value was measured to be 1.12, it was extremely enough to constitute interaction molecular chemical energy to initiate receptor-donor activation and signal transduction. The mono-Protease and dynamic-enzyme inhibitor values were found to be 0.77 and 0.15 respectively in the present case. Due to those moderate coefficients of Protease and enzyme inhibitors, the present drug acts as a competitive catalyst to assist the conversion of enzyme's substrates into products [19].

### 4.4 Vibrational representation

#### 4.4.1 Vibrational task

**Figure 5:**
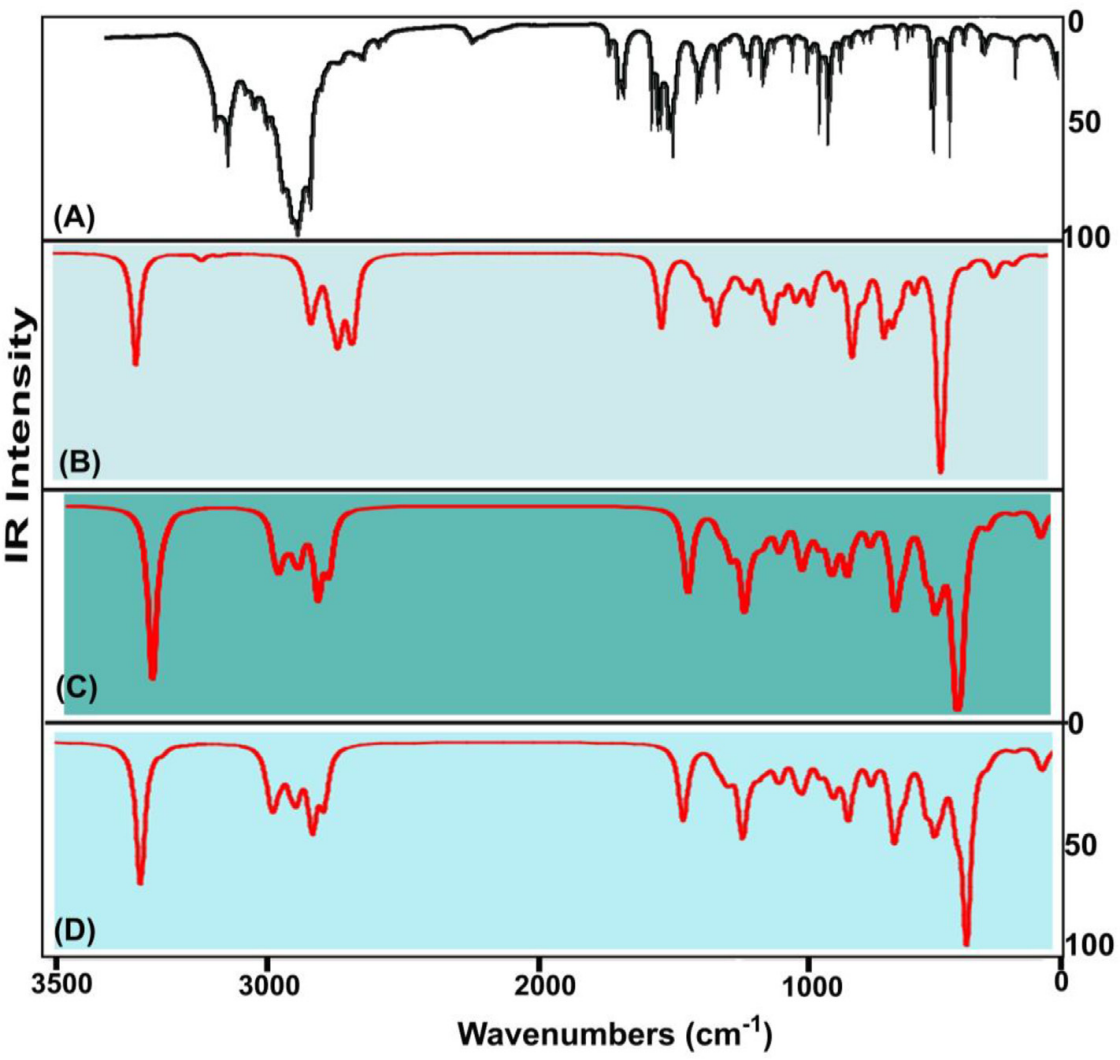
(a) Experimental (b) HF(c) B3LYP (d) B3PW91 FT-IR spectra of 2-Amino-1-phenyl-1-propanol.

**Figure 6:**
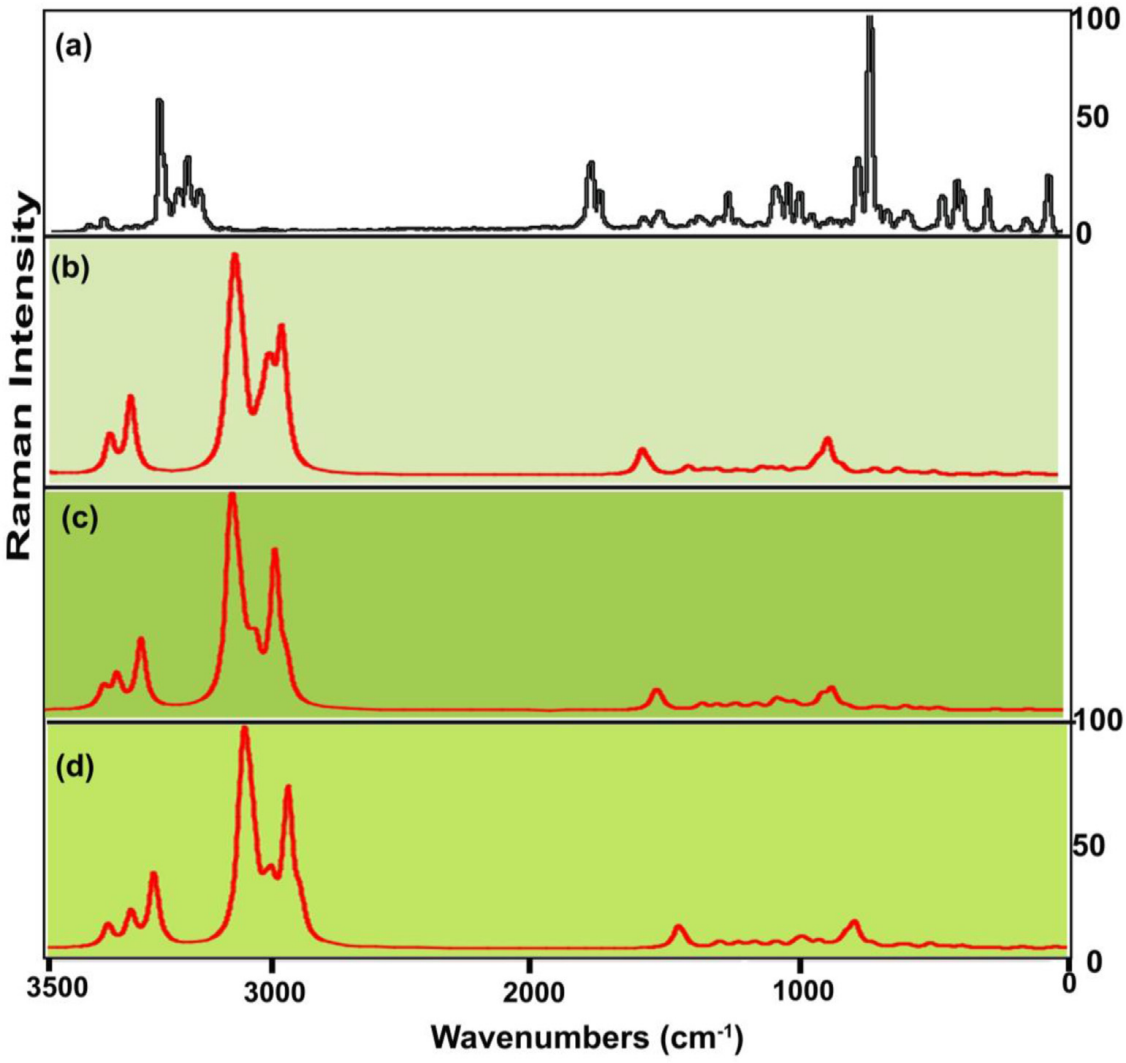
(a) Experimental (b) HF(c) B3LYP (d) B3PW91 FT-Raman spectra of 2-Amino-1-phenyl-1-propanol.

The combined FT adopted IR and Raman spectra of finger print and group frequencies are displayed in Figures 5 and 6 and their corresponding wavenumbers plotted in Table 3. According to the irreducible representation in group theory, the symmetry of C_S_ point group was assigned, thereby all the vibrations are divided to be in-plane and out-plane vibrations; such as represented as A′ and A″. These vibrations have been calculated and arranged using the formula; Total vibrational motion = 3N-6 in which N- stretching, N-3 in-plane and N-3 out-of-plane bending vibrations. All vibrational bands would have been configured as 66 vibrations = 45A′+21A″

**Table 3:** Experimental and calculated vibrational frequencies of 2-Amino-1-phenyl-1-propanol.

Symmetry Species C_s_	Observed Frequency (cm-^1^)		Methods					Vibrational Assignments
			HF	B3LYP		B3PW91		
	FT-IR	FT-Raman	6-311++G (d, p)	6-31+G (d, p)	6-311++G (d, p)	6-31++G (d,p)	6-311++G (d, p)	
A′	3400w	-	3600	3598	3610	3589	3609	(O–H) υ
A′	3280vs	-	3496	3478	3461	3472	3488	(N–H) υ
A′	3250vs	-	3450	3456	3449	3444	3440	(N–H) υ
A′	-	3055m	3064	3070	3054	3047	3066	(C–H) υ
A′	3050vs	-	3046	3057	3043	3037	3056	(C–H) υ
A′	-	3020vw	3014	3014	3031	3024	3009	(C–H) υ
A′	3000vs	-	3003	3004	3007	3014	2999	(C–H) υ
A′	-	2990vw	2991	2990	3001	2998	2984	(C–H) υ
A′	2950vs	-	2942	2957	2951	2946	2957	(C–H) υ
A′	2900vs	2900w	2914	2903	2900	2895	2905	(C–H) υ
A′	2850vs	-	2870	2845	2850	2858	2844	(C–H) υ
A′	2800s	2800 w	2806	2802	2796	2800	2807	(C–H) υ
A′	2795s	-	2696	2701	2702	2696	2712	(C–H) υ
A′	1620m	-	1620	1614	1624	1621	1617	(C=C) υ
A′	-	1610w	1605	1610	1615	1607	1604	(C=C) υ
A′	1600s	-	1593	1605	1595	1601	1597	(C=C) υ
A′	1500vs	-	1490	1505	1498	1506	1502	(C–C) υ
A′	-	1470vw	1460	1467	1475	1474	1470	(C–C) υ
A′	1450vs	-	1445	1441	1453	1452	1449	(C–C) υ
A′	1440m	-	1442	1446	1438	1445	1442	(O–H) δ
A′	1390s	-	1399	1396	1398	1394	1398	(N–H) δ
A′	1385s	1385vw	1395	1392	1391	1393	1373	(N–H) δ
A′	1370w	-	1375	1371	1369	1369	1366	(C–C) υ
A′	1350w	-	1355	1348	1347	1353	1346	(C–C) υ
A′	1330s	-	1322	1329	1331	1332	1328	(C–C) υ
A′	-	1290vw	1292	1288	1293	1285	1285	(C–H) δ
A′	1260w	-	1261	1260	1255	1262	1258	(C–H) δ
A′	1200s	-	1197	1201	1239	1204	1201	(C–H) δ
A′	-	1180w	1180	1182	1184	1185	1182	(C–H) δ
A′	-	1160vw	1167	1155	1155	1162	1162	(C–H) δ
A′	1140m	-	1142	1144	1143	1143	1141	(C–H) δ
A′	-	1130vw	1126	1130	1128	1129	1126	(C–H) δ
A′	1090m	-	1078	1093	1091	1091	1088	(C–H) δ
A′	-	1060vw	1065	1057	1056	1066	1062	(C–H) δ
A′	1050vs	-	1048	1053	1049	1048	1046	(C–H) δ
A′	1030vs	-	1038	1034	1032	1029	1027	(C–N) υ
A′	1015s	-	1011	1014	1018	1013	1019	(C–O) υ
A″	-	1010vs	998	1010	1011	1010	1010	(N–H) γ
A″	-	1000vs	995	1003	1001	996	996	(N–H) γ
A″	980w	-	993	979	975	976	977	(C–H) γ
A″	-	960vw	959	961	960	959	962	(C–H) γ
A″	-	940vw	955	938	935	936	945	(C–H) γ
A″	930m	-	919	930	929	910	930	(C–H) γ (CCO) υ
A″	-	860vw	850	855	863	858	857	(C–H) γ
A″	850w	-	849	846	854	848	848	(C–H) γ
A″	820w	-	818	823	821	820	818	(C–H) γ
A″	-	800vw	803	796	796	803	801	(C–H) γ
A″	745vs	-	744	743	742	728	746	(C–H) γ
A″	700vs	-	700	702	709	703	704	(C–H) γ
A″	680w	-	679	678	702	681	681	(O–H) γ
A′	-	610vw	608	626	612	610	609	(C–N) δ
A′	600w	-	598	612	602	601	602	(C–O) δ
A′	520w	-	519	598	520	520	520	(CCC) δ
A′	500w	-	502	499	499	499	487	(CCC) δ
A′	-	420vw	421	419	420	418	421	(CCC) δ
A″	-	400vw	401	398	401	399	399	(C–N) γ
A″	-	380vw	381	380	381	381	379	(C–O) γ
A″	300w	-	299	307	301	300	301	(CCC) γ
A″	-	295vw	294	296	296	294	296	(CCC) γ
A″	-	280w	280	280	281	279	281	(CCC) γ
A′	-	250vw	249	250	250	250	250	(C–C) δ
A′	-	220vw	220	220	220	220	220	(C–C) δ
A′	200w	-	200	200	200	201	201	(C–C) δ
A″	100w	-	100	100	100	100	100	(C–N) γ
A″	70w	-	85	70	70	70	70	(C–C) γ
A″	44w	-	36	44	44	36	44	(C–C) γ

VS –Very strong; S – Strong; m- Medium; w – weak; as- Asymmetric; s – symmetric; υ – stretching; α –deformation, δ - In plane bending; γ-out plane bending; τ – Twisting.

All the vibrational bands are noted according to their characteristics vibrational region, here, the O–H vibrational bands were leaded all the other vibrational bands and followed by N–H vibrational frequencies and the ring breathing modes were completed the end of vibrational zone.

#### 4.4.2 CH–O–H vibrational process

The first part of the chain was the CHOH group which initiated the substitutional effect in the molecule. This group was so sensitive since the hydroxyl group was attached with the aliphatic group. Here, the O–H stretching band, in-plane sequence and wagging bands are assigned in the region of 3300-3150 cm^−1^, 1430-1370 cm^−1^ and 660-600 cm^−1^ respectively [20]. For this case, all were determined at 3400, 1440 and 680 cm^−1^ correspondingly. All those vibrational bands found well above the expected wavenumber boundary which was due to the energy support of the CH group. The C–O stretching, rocking modes and wagging is generally identified in the finger print region 1150-1075 cm^−1^, 500-440 cm^−1^ and 390-330 cm^−1^ [[21], [22]] respectively. In addition to that, the CCO stretching is usually assigned in the region 900-800 cm^−1^ [23]. But, here, the C–O stretching, C–O bending modes were observed at 1015, 600 and 380 cm^−1^ respectively. The CCO stretching band was recognized at 930 cm^−1^. All these vibrational signals observed strongly and within the allowed spectrum region which represents their presence and strong involvement in the molecular property. For the aliphatic chain, the C–H stretching and bending modes are to be allotted at the vibrational region 2935-2840 cm^−1^, 1240-890 cm^−1^ and 900-675 cm^−1^ [24] in order. Here, the stretching and bending signals are found at 2795, 1050 and 700 cm^−1^ respectively. High energy vibrations are only elevated well above the allotted limit whereas the low energy bands were found within the limit. Such an elevation in observed bands was mainly by the exchange of interactive chemical potential among groups.

#### 4.4.3 CH–NH vibrations

The second segment of substitutions in the ring was CHNH_2_ group and they are coupling of CH and NH groups which acted intermediate chemical stimulator on chain. The N–H vib-stretching, vib-bending modes are usually observed in the group vibrations zone 3500- 3300 cm^−1^, 1590-1500 cm^−1^ and 1020-950 cm^_1^ in order [25]. For the present case, the stretching, in-plane (scissoring) and out-of-plane (Wagging) bending modes were recognized at 3280 & 3250 cm^−1^, 1390 & 1295 cm^−1^ and 1010 & 1000 cm^−1^ in that order. Entire vibrational bands were appeared below the expected zone which was due to the energy sharing with the C–H bond in order to equalize the chemical interactions. Usually, this band is dominated all the vibrations since it has force constant with higher order. But here, the chemical related energy of N–H was exchanged to the main part of the substitutions. This clearly showed that, part of NH potential was shared to hold the CH entity. The linked C–H vibrations are ordered in the vibrational zone for stretching and bending are 2820-2740 cm^−1^, 1240-890 cm^−1^ and 900-675 cm^−1^ correspondingly [26]. In this case, the C–H bond stretching, rocking and wagging or twisting vibrations were determined at 2800, 1060 and 745 cm^−1^ respectively. Such vibrations were packed within the prearranged characteristics region and here the chemical restored energy of C–H bonds were sustained which was by the support of N–H bond.

#### 4.4.4 C–H vibrations

Usually, the aromatic class derivatives, C–H vibrations are mostly influenced by the allied core and chain vibrations [27]. These vibrations for aromatic cyclic compound are typically observed in the region 3100-3000 cm^−1^ (stretching), 1300-1000 cm^−1^ (in-plane bending) and 1000-750 cm^−1^ (out-of-plane bending) [28]. For this compound, the stretching was observed at 3055, 3050, 3020, 3000 and 2990 cm^−1^, the in plane bending was found at 1290, 1260, 1200, 1180 and 1160 cm^−1^ and out of plane bending modes identified at 980, 960, 940, 930 and 860 cm^−1^ respectively. Here, irrespective of stretching bands and I/P-bending modes, all the vibrational bands were found to be well within the expected region. This ensured the exchange of chemical energy from chain to ring unidirectional.

#### 4.4.5 Core vibrations

Usually the core vibrations such as CC and CCC stretching and bending vibrational peaks are purposively influenced due to the injection of substitutional groups. Those vibrational regions were disturbed abruptly with respect to the mass and electronegativity of the substitutions [29]. The core C=C and C–C stretching signals in general they assigned in the region 1630-1450 cm^−1^ [30]. Here, in the molecule, the C=C and C–C stretching modes were traced at 1620, 1610 & 1600 cm^−1^ and 1500, 1470 and 1450 cm^−1^ in order. In this case, no increment and decrement was observed in the ring stretching modes which repeatedly ensured the energy flow from chain to ring. As usual, according to the above literatures, the ring breathing modes are limited in the wavenumber region 620-410 cm^−1^ and 400-310 cm^−1^. As per the Table 3, the in-plane and out-of-plane breathing modes were observed at 520, 500 & 420 cm^−1^ and 300, 295 & 280 cm^−1^ respectively. The low energy related vibrations also have not been affected by the chain vibrational energy. The centre of symmetry of the molecule is focused on the bridge point of the chain and ring by which the existence interactive forces between electronegative and protonic contents are balanced. The total vibrational energy potential of chain was found to be equal to ring and thus the oscillation of chemi-potential taking place between two segments of the molecule.

#### 4.4.6 CH_3_ vibrations

Usually, the methyl group vibrations are disturbed due to the attachment of the adjoining ligand groups. Here, the methyl group was injected at the end of the chain and this was the starting point for the chemical energy oscillation [31]. According to the literature [32], the C–H vib-stretching, rocking and wagging vibrations are observed in the region 2975-2920 cm^−1^ (asymmetry stretch) & 2870-2840 cm^−1^ (symmetry stretch), 1250-940 cm^−1^ and 910-680 cm^−1^ respectively. In this case, the stretching bands were found at 2950, 2900 and 2850 cm^−1^, in plane bending modes were spotted at 1140, 1130 and 1090 cm^−1^ and out of plane bending was organized at 850, 820 and 800 cm^−1^ respectively. All those vibrations measure to be within the expected region of spectrum which clearly inferred that, the vibrational region energy was boosted up by the support of other groups in chain. Thus, the CH_3_ have additive energy to exchange vib-chemical potential to the base-ring.

### 4.5 NMR analysis

**Figure 7:**
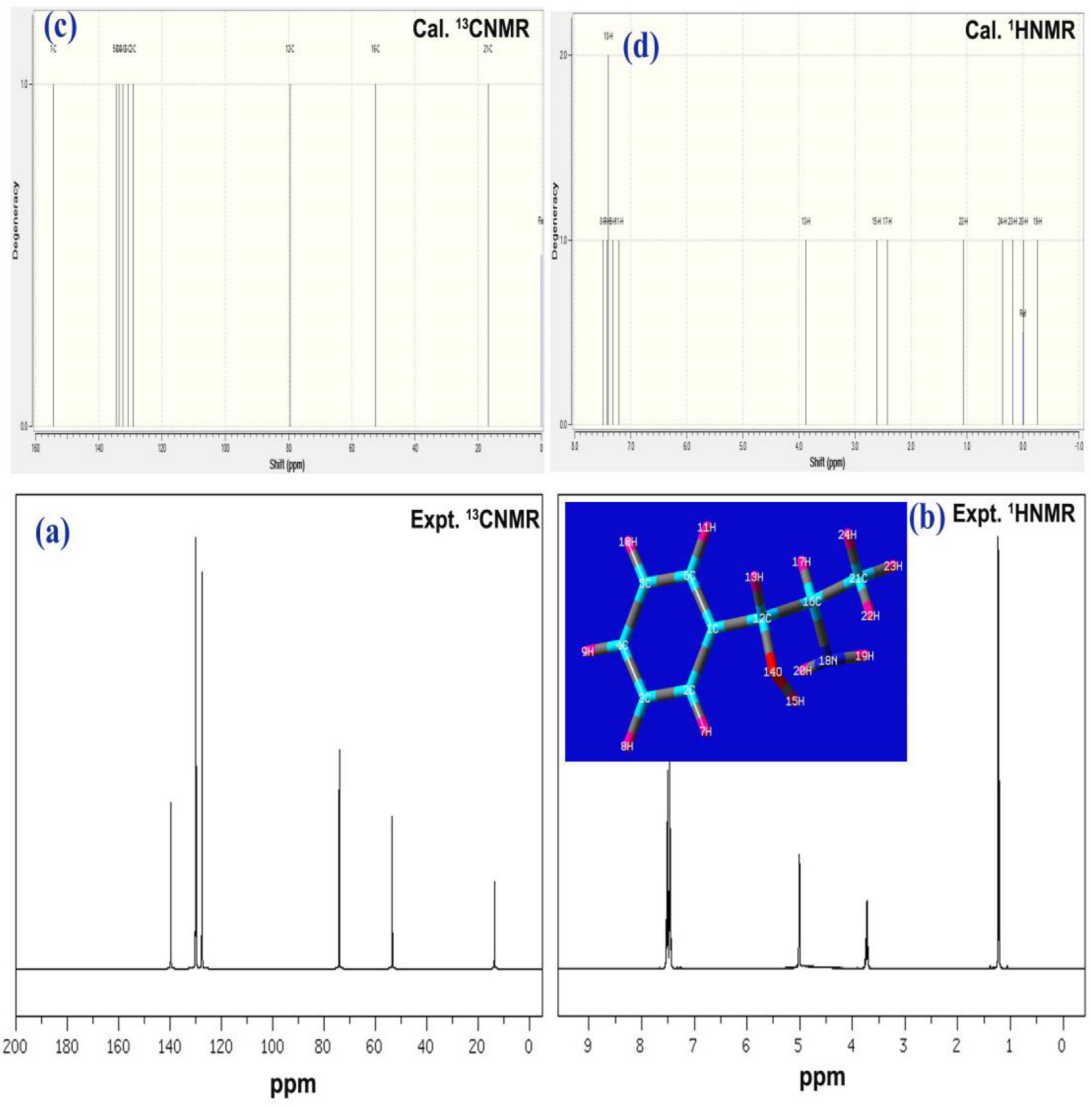
(a and b) Experimental ^13^C NMR ^1^H NMR (c and d) Theoretical ^13^C NMR ^1^H NMR spectra of 2-Amino-1-phenyl-1-propanol.

The NMR chemical shift was portrayed in Table 4 and the corresponding spectra are presented in Figure 7. The chemical reaction path mechanism is internally made on the molecule by ordering the chemical shift for inducing a chemical environment to set up the required chemical potential. Thus, the architected potential through the de-shielding effect on main core and allied carbons are oscillated to achieve the drug activity. In this case, they were found to be ordered from C1 to C6 and C5 and from C1 to C2 and C3. According to the chemical shift occurred in the molecule, the base ring carbons were sequenced as C1>C5>C3>C4>C6>C2 among which the organized chemical potential from chain was oscillated and the resultant chemi-drug potential was formed. The main nodal carbon; C1 was chemically shifted to be 154 ppm (expt. = 152 ppm) on which the entire oscillation was taking place.

**Table 4:** Experimental and calculated ^1^H and ^13^C NMR chemical shift of 2-Amino-1-phenyl-1-propanol.

Atom position	Chemical Shift - TMS-B3LYP/6-311+G(2d,p) (ppm)			Experimental shift (ppm)
	Gas	Solvent phase		
		DMSO	CCl_4_	
C1	154.43	153.90	154.21	152
C2	129.18	128.92	129.14	128
C3	133.42	133.77	133.57	134
C4	132.16	132.64	132.34	134
C5	134.27	134.72	134.42	134
C6	130.52	131.20	130.73	134
C12	79.59	79.32	79.47	60
C16	52.49	52.23	52.40	48
C21	17.0	16.49	16.78	15
H7	7.37	7.47	7.41	8.0
H8	7.42	7.58	7.49	8.0
H9	7.24	7.41	7.31	-
H10	7.32	7.50	7.39	-
H11	7.13	7.35	7.21	-
H13	3.79	3.99	3.87	3.7
H15	2.48	2.76	2.61	2.4
H17	2.37	2.507	2.41	2.4
H19	-0.42	-0.020	-0.257	-
H20	-0.027	0.014	-0.010	-
H22	1.182	0.854	1.065	-
H23	0.12	0.2891	0.183	-
H24	0.32	0.4281	0.364	-

In the substitutional chain, the chemical shift of allied carbons C12, C16 and C21 was measured to be 79, 52 and 16 ppm. Here, the shift was more in C12 than the rest of two which was due to the electronic domain was partially transferred from C16 and C21. This process happened in a unidirectional way, i.e. from C21 of CH_3_ to C12 of CHOH group. Though, the carbons C12 and C16 were injected by hydroxyl and amino group (highly electronegative) the required electron cloud were sustained on the carbons and due to the paramagnetic shielding the chemical shift was decremented to protect the carbons (nodal points) to stream out dynamic-chemi potential to ring. In the case of H in the core ring, the chemical-shift of all was observed uniformly and there was no influence over that was found. But, in the case of H in CHOH, CHNH and CH_3_ places, the random chemical shift was observed which showed the great influence taking place on such groups in order to prepare the drug active potential.

### 4.6 Frontier energy interaction analysis

The electronic configuration is completely altered on par with the chemical equivalent interactions generated on the atoms and such electronic orbitals in different levels are split up occupied and unoccupied orbital segments. The clusters of transitions among different degenerate and non degenerate energy levels induced various domains of chemical kinetics while absorbing external energy. By measuring chemical equivalent energy calculated from interactive orbitals, the chemical potential can be found which organizes molecular properties that decide drug activity [33].

**Figure 8:**
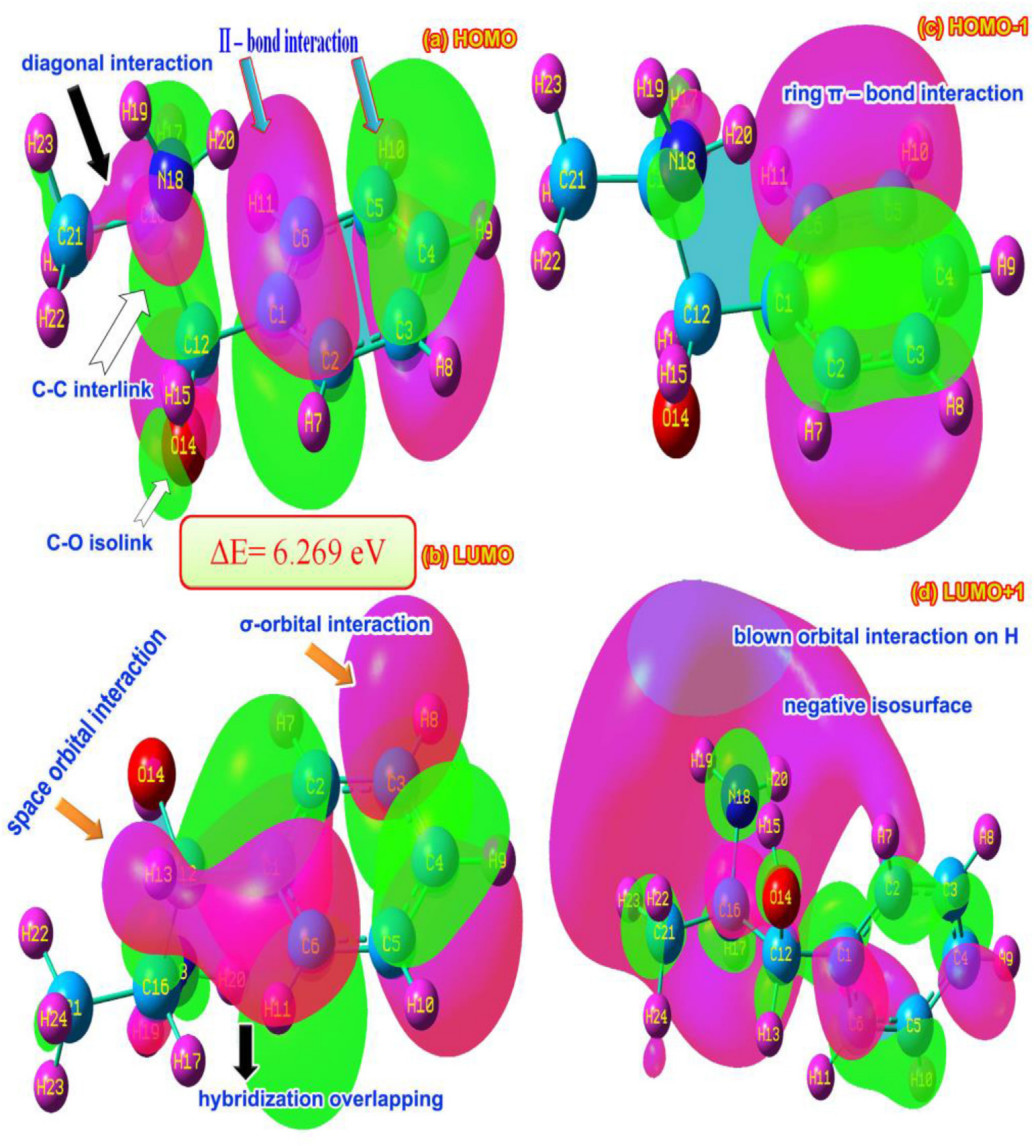
(a) HOMO (b) LUMO(c) HOMO-1 (d) LUMO-1 of 2-amino-1-phenyl-1-propanol.

The frontier molecular space and interactive orbital lobes are drawn using chemical field distribution grid points which shown are in Figure 8 and energy levels shown in Table 5. The frontier molecular system is characterized by σ, π and δ bonding orbital interactions which are taking place by existence of molecular arrangement. The HOMO was represented by semicircle CCC π-bindings and they were segmented into two parts in which the characteristics were restricted with respect to orbital interaction. Since both the iso surface was reversed in the ring, the positive and negative lobes were found oppositely. The interactions were extended from C16 to H21 and H23 due to the degenerate orbital overlapping. The degenerate interactive orbitals found between C12, O14 and H15 and C12 and C16. Thus such arrangement was mingled with NH_2_ lobe interactions and this formation of overlapping showed the cluster of electron domain was about to transfer in order to prepare drug property.

**Table 5:** Frontier molecular orbitals with energy levels of 2-Amino-1-phenyl-1-propanol.

Energy levels	IR region	UV-Visible region
	B3LYP/6311++G(d,p) Energy (eV)	B3LYP/6311++G(d,p) Energy (eV)
H+10	10.7009	10.6334
H+9	10.5463	10.4786
H+8	10.2878	10.2350
H+7	9.9610	10.0981
H+6	9.5273	9.7224
H+5	9.4459	9.5156
H+4	8.9354	9.0219
H+3	7.6508	7.5982
H+2	7.2129	7.1626
H+1	7.0388	7.0175
H	6.8211	6.8763
L	0.5516	0.6917
L-1	0.4648	0.6196
L-2	0.3831	0.3663
L-3	0.0376	0.0299
L-4	0.0931	0.0563
L-5	0.3804	0.4142
L-6	0.5483	0.5260
L-7	0.6485	0.6001
L-8	0.8479	0.9246
L-9	1.0436	1.1053
L-10	1.2313	1.1627

In LUMO, single σ and π bonding interaction was found over C–H and C–C of hexagonal core in which the empty orbitals were found to accept the transitional electrons to consume chemical energy to attach the drug energy. One space orbital interaction appeared between C6 and H13 of aromatic and aliphatic which ensured blending of orbitals that were empty to receive electron clouds. Another π-interacting bonding system was found between C2 and C1 (H7) of core and C16 of CHOH group and this arrangement was found to be formed the centralized orbital system to receive similar energy electron. The LUMO+1 (second order interactive system) orbital interaction appeared on aromatic and aliphatic C and the blown orbital interaction was found over all the H of the core and allied C–H. In second order HOMO-1, the transitions electron cloud disappeared and fully migrated to aromatic ring CC, in which the π-bonding interaction profile was seen. In two extreme ends (HOMO-1to LUMO+1), the core and aliphatic chain was occupied vice versa. From this observation, it was observed that the chemical energy gap was found to be 6.269 eV which showed very high chemical inertness and very low softness.

### 4.7 CT-complex analysis

**Figure 9:**
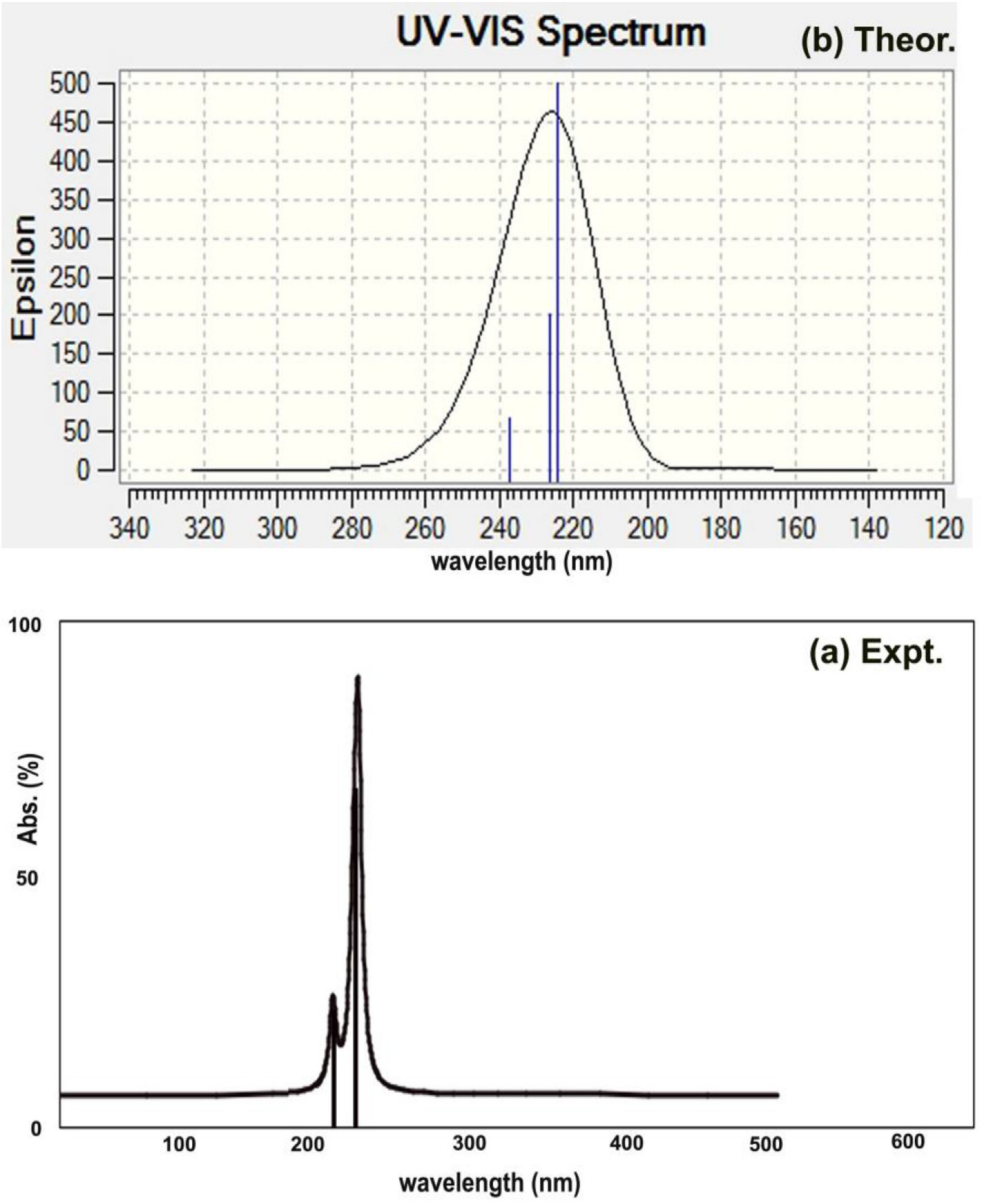
(a) Experimental (b) Theoretical UV-Visible spectra of 2-Amino-1-phenyl-1-propanol.

UV-Visible spectra were obtained from electronic transitions taking place between the electronic energy levels which comprised of vibrational energy states. The entire vibrational characteristics can be understood by observing electronic spectra in which the CT complex species can be identified from the absorption peak [34]. The experimental and theoretical absorption peaks for UV-Vis is presented in Figure 9 and the corresponding parameters are depicted in Table 6.

**Table 6:** Electronic spectral parameters of 2-Amino-1-phenyl-1-propanol.

λ (nm)	E (eV)	(*f*)	Transition Level	Major contribution	Assignment	Region	Bands
**Gas**							
237.25	5.2258	0.0018	H+2→L-1 (43%)	H-1→L (56%)	n→π∗	Quartz UV	R-band (German, radikalartig)
226.17	5.4819	0.0034	H+1→L (39%) H→L (46%)				
224.09	5.5328	0.0070	H→L-1 (56%)				
**DMSO**							
236.62	5.2399	0.0023	H+2→L-1 (44%)	H→L-1 (57%)	n→π∗	Quartz UV	R-band (German, radikalartig)
224.81	5.5150	0.0038	H+1→L (38%) H→L (47%)				
223.22	5.5543	0.0120	H→L-1 (57%)				
**CCl**_**4**_							
237.16	5.2278	0.0027	H+2→L-1 (43%)	H→L-1 (56%)	n→π∗	Quartz UV	R-band (German, radikalartig)
225.38	5.5010	0.0043	H→L (46%)				
223.61	5.5447	0.0116	H→L-1 (56%)				

H: HOMO; L: LUMO.

The observed doubly degenerated electronic peaks were found to be located at 220 and 230 nm whereas the theoretical absorption peaks were identified at 224 and 226 nm along with small excited peak at 238 nm at energy gap of 5.2, 5.4 and 5.5 with oscillator strength (0.001, 0.003 and 0.007). Such transitions were assigned to H to L, H-1 to L and H to L+1 energy levels which were represented by n→π∗ transition profile and belong to Q-UV zone of spectrum (R-band (German, radikalartig)). In solvent condition, the peaks were determined at 223, 224 and 236 nm at 5.2, 5.5 and 5.5 respectively with oscillator strength of 0.002, 0.003 and 0.01 which were assigned to Quartz UV region of spectrum (n→π∗ transition state). From this observation, it was inferred that, all the peaks were observed with little fluctuations. In this molecule, two active and one passive ligand groups were identified and among which the charge transfer complex to be identified. As per the electronic absorption peak at particular wavelength and selection rule, the CT complex was found to be C1–C12 with C16–N18 which are doubly degenerate and form n→π∗ transition. These two n→σ∗ transitions combined together and formed same degenerate level profile produced resultant n→π∗.

### 4.8 Physico-chemical parameters

In order to obtain physico-chemical parameters, the structural optimization is very important and it can be calculated from the geometrical energy at zero vibrational state. It was 480 Hartree in both free-IR and restrict-UV-Visible regions which showed optimization of present structure. The electron affinity and low-ionization potential was determined to be 6.8 and 0.55 eV respectively. Here, the electron affinity was high which showed electron rich zones around the molecule that can bind with protein in any angle of the plane. The ionization potential was very low and denoted the present molecule to be highly covalent character. The Physico-chemical parameters of 2-Amino-1-phenyl-1-propanol are portrayed in Table 7.

**Table 7:** Physico-chemical parameters of 2-Amino-1-phenyl-1-propanol.

Parameter	IR region	UV-Visible region	Electrophilicity charge transfer (E_CT_) (ΔN_max_)_A_-(ΔN_max_)_B_
E_total_ (Hartree)	-480.899	-480.695	
E_HOMO_ (eV)	6.8210	6.876	
E_LUMO_ (eV)	0.5515	0.6917	
ΔE_HOMO-LUMO gap_ (eV)	6.2695	6.1846	
E_HOMO+1_ (eV)	7.0387	7.0175	
E_LUMO-1_ (eV)	0.4647	0.6196	
ΔE_HOMO-1-LUMO+1 gap_ (eV)	6.5740	6.3979	+0.0685
Chemical hardness (η)	3.1347	3.0923	
Electronegativity (χ)	-3.686	-3.7840	
Chemical potential (μ)	6.2695	6.1846	
Chemical softness(ξ)	0.1595	0.1617	
Electrophilicity index (ψ)	2.1675	2.315	
Dipole moment	2.948	2.675	
E_CT_	2.4720	1.304	

Unless the molecule possesses chemical hardness, its reaction capability becomes weak and reacts with any molecule easily and it needs more binding energy coupled with protein. Here, the values were measured as 3.13 and 3.09 in both regions which are deliberately so high enough to sustain its chemical stability. The aromatic compound should have chemical softness more than unity and the same was found to be 0.15 and 0.16 respectively in both regions. For this case, it should be more than unity and it was the main drawback for the present compound. The electronegativity was as 3.6 and 3.7 correspondingly for IR and UV-Visible region which was very high due to the considerable depletion reinforcement of the negative atoms and it was good for the present drug species. The Electrophilicity index for the title compound was to be 2.16 and 2.31 in order for IR and UV region. This coefficient showed the well dispersion characteristics of electron clouds and it was so better to understand the antisymmetry rate of chemical species to recognize drug properties. The dipole moment was found to be 2.94 and 2.67 which exposed that the present case would have proper asymmetrical charge dislocation for generating binding affinity with protein. The E_CT_ of the present molecule was determined to be 2.47 and 1.30 respectively for the IR and UV region which showed the specific hyper polarization of the present chemical species. This was more in IR than UV-Visible and inferred that the present case is more reactive than UV-Visible. So, it can be influenced by heat rather than light. The Electrophilicity charge transfer was determined to be +0.0685 and this value demonstrated the chemical interactive energy was transferred from chain to ring for assembly required chemical potential for drug activity.

### 4.9 Biological hyper activity analysis

The pre-assignment of charge levels on the molecular sites on compound are usually estimated in variable order; first and second order. Normally, the first of hypo-order is measured valance banded electron delocalization and were occupied with high electro-motive generated by interactive forces on direct molecular sites and furnish static chemi-potential for modifiable drug activity. In the second or hyper-order, the interactive zone electrons with strong binding energy with nucleus delocalized with frenkel defective forces generates enforced asymmetrical scattering-polarization called hyperpolarisation resulting intra atomic static potential causing sensitive drug commotion. All the polarization parameters in different coordinates are illustrated in Table 8.

**Table 8:** The Polarizability α(a.u.) and the first hyperpolarizability β(esu) of 2-Amino-1-phenyl-1-propanol.

parameters	B3LYP/6–31++G(d,p)
α_xx_	-62.93
α_xy_	-3.476
α_yy_	-66.65
α_xz_	-2.24
α_yz_	-3.57
α_zz_	-69.12
α_tot_	146.97
Δα	196.36
μ_x_	-0.341
μ_y_	2.0321
μ_z_	2.164
μ_tot_	2.988
β_xxx_	-5.58
β_xxy_	4.61
β_xyy_	4.83
β_yyy_	-3.50
β_xxz_	15.66
β_xyz_	0.793
β_yyz_	2.630
β_xzz_	-15.28
β_yzz_	3.923
β_zzz_	10.69
β_tot_	89.581

For the present case, the average and total polarization coefficients were noted to be 146.9 × 10^−33^ esu and 196.3 × 10^−33^ esu respectively. Here the hypo-order polarization on chain and ring was enabled and causing strong drug hardness capability. The second order polarization was calculated with respect to three internal coordinates and is found to be 89.581 × 10^−33^ esu for this case. In this molecule, the hyper-order polarizability of the compound was enabled strongly and showed interactive-orbital stabilization fascinated for consistent drug potential. By such observation, it can be noted that this compound can be modified anyway by adoption of substitutional groups for inductive multifunctional drug movement.

### 4.10 MEP analysis

**Figure 10:**
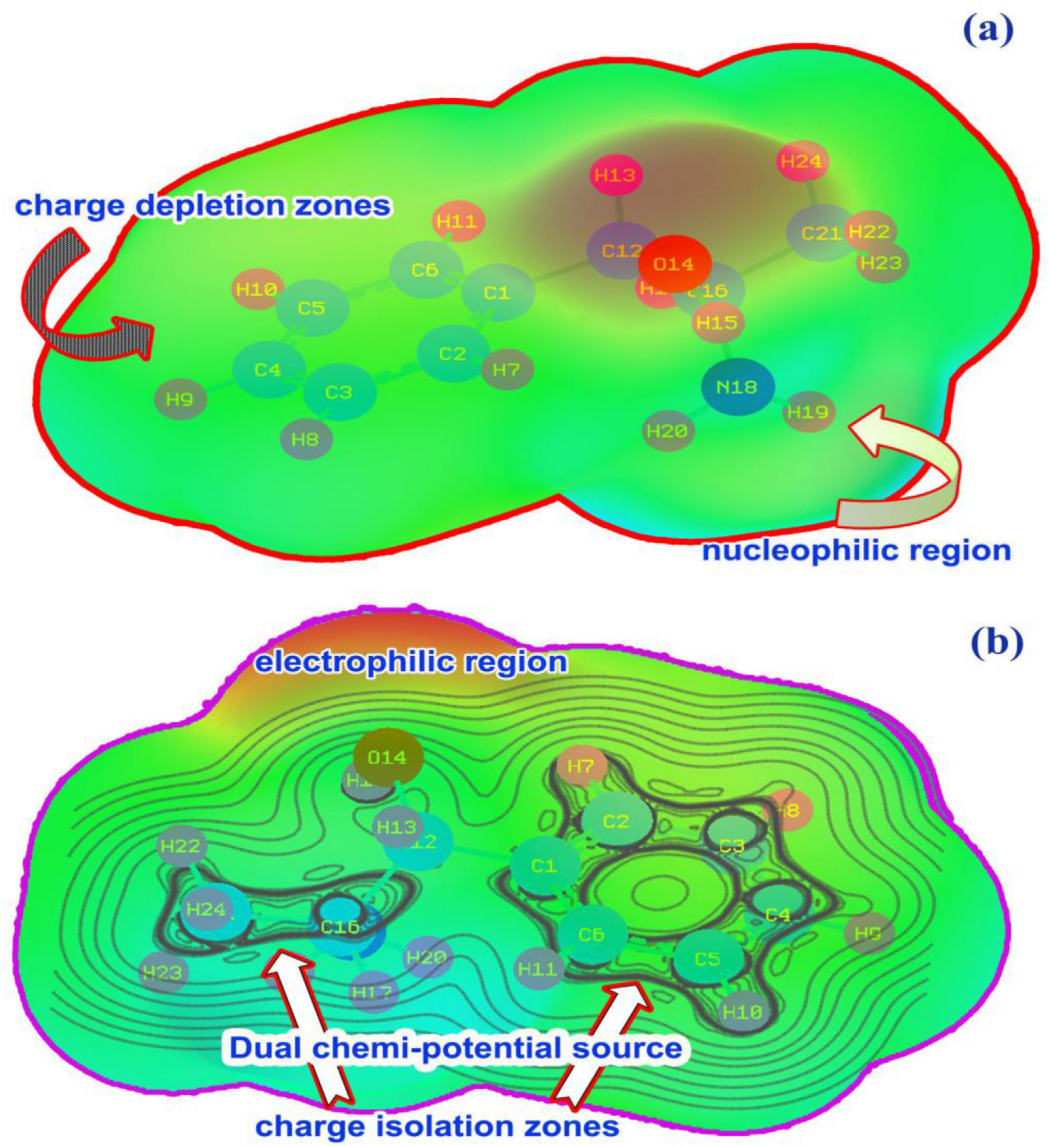
(a) MEP (b) MEP contour of 2-Amino-1-phenyl-1-propanol.

**Figure 11:**
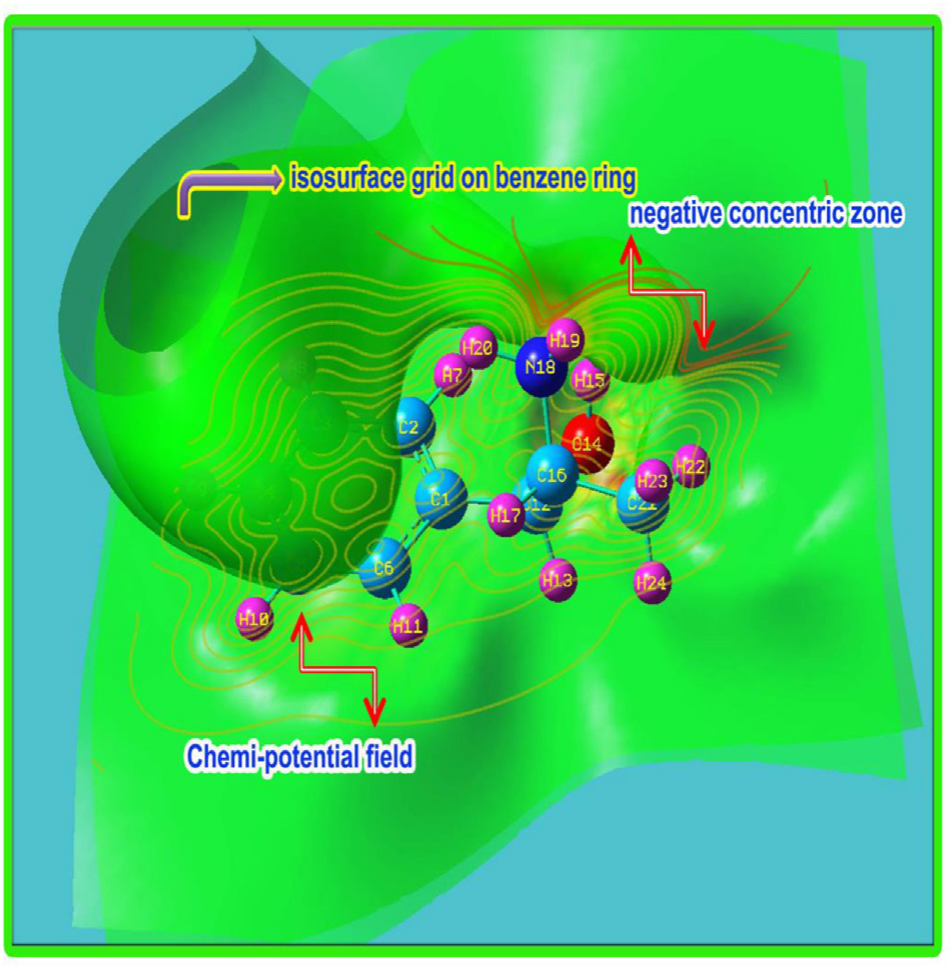
Chemi-potential contour of 2-Amino-1-phenyl-1-propanol.

Usually, the molecular static-electro potential is mainly applied for measuring the charge depletion rate in molecular site which is linked with protein binding capability. The depletion rate is usually measured by colour gradient around the molecular entities. The entire molecular electrostatic potential surface was specifically drawn using isosurface grid points which associated with potential field distribution as shown in Figures 10 and 11 respectively. Here, the high degree of electrophilic region was seen over H–O bonds, especially it should be located on H–N–H bonds since the high energy positive species created on NH groups. But, here, the resultant electrophilic zone was shifted to the O–H bonds which were observed to be high than other negative affiliated entities.

The protonic region called nucleophilic region in which the species are more protonic affinity and they were appeared over H–N–H bonds. The protonic zones were to be observed on CH_3_ group in this case because the CH_3_ has rather electronic accumulated zone where the protons were redirected. The migrated positive loving zones were also found around the ring C–H and due to the partial charge delocalization on allied C–H bonds around the molecule, the intermediate zones were identified on the C–H bonds. In this case, dual potential sources were recognized on ring and NHCH_3_ groups from where, the chemical potential field was originated and dispersed around the molecule. This makes high potential forceful asymmetrical charge flow which generates hyperactive pressure inside the molecular entities causing antifungal drug potential. The Figure 10 also showed chemi-potential field distribution among the molecular site and iso surface grid valley focused on centre of the hexagonal ring and it was splitted according to the centre of symmetry which was located on CHNH group.

### 4.11 Non bonding molecular transitions analysis

The chemical energy in terms of electronically induced transitions between the molecular bonding and antibonding orbitals described the chemi-equipotential storage for preparing drug mechanism process [35]. The non bonded orbitals usually exchange the chemic-potential by means of electronic energy by making transitions to antibonding orbitals which is normally measured in terms of eV and kcal./mol using non perturbation molecular arrangements. The measured non bonded electronic energy levels are illustrated in Table 9.

**Table 9:** The calculated NBMO of 2-Amino-1-phenyl-1-propanol by second order Perturbation theory.

Donor (i)	Type of bond	Occupancy	Acceptor (j)	Type of bond	E2 kcal/mole	Ej – Ei au	F(I j) au
**C1 – C2**	π	1.97356	**C1 – C6**	π∗	3.67	1.27	0.061
**ˮ**	π		**C2 – C3**	π∗	3.07	1.27	0.056
**ˮ**	π		**C3 – C4**	π∗	21.02	0.28	0.069
**ˮ**	π		**C5 – C6**	π∗	20.14	0.28	0.067
**C1 – C6**	σ	1.97436	**C1 – C2**	σ ∗	3.60	1.28	0.061
**ˮ**	σ		**C5 – C6**	σ ∗	3.19	1.28	0.057
**C1 – C12**	σ	1.97400	**C2 – C3**	σ ∗	2.40	1.20	0.048
**ˮ**	σ		**C5 – C6**	σ ∗	2.41	1.20	0.048
**C2 – C3**	σ	1.97879	**C1 – C2**	σ ∗	3.52	1.28	0.060
**ˮ**	σ		**C1 – C12**	σ ∗	3.51	1.10	0.056
**C2 – H7**	σ	1.97836	**C1 – C6**	σ ∗	4.89	1.08	0.065
**ˮ**	σ		**C3 – C4**	σ ∗	3.70	1.09	0.057
**C3 – C4**	π		**C1 – C2**	π∗	19.31	0.29	0.067
**C3 – C4**	π		**C5 – C6**	π∗	20.84	0.28	0.068
**C3 – H8**	σ	1.98107	**C1 – C2**	σ ∗	3.83	1.10	0.058
**ˮ**	σ		**C4 – C5**	σ ∗	3.73	1.09	0.057
**C4 – H9**	σ		**C5 – C6**	σ ∗	3.77	1.10	0.058
**ˮ**	π	1.97895	**C1 – C6**	π∗	3.58	1.27	0.060
**ˮ**	π		**C1 – C12**	π∗	3.35	1.11	0.055
**ˮ**	π		**C1 – C2**	π∗	20.71	0.29	0.070
**ˮ**	π		**C3 – C4**	π∗	19.39	0.28	0.067
**C5 – H10**	σ	1.98123	**C1 – C6**	σ ∗	3.87	1.09	0.058
**ˮ**	σ		**C3 – C4**	σ ∗	3.62	1.10	0.056
**C6 – H11**	σ	1.97891	**C1 – C2**	σ ∗	4.67	1.10	0.064
**ˮ**	σ		**C4 – C5**	σ ∗	3.71	1.10	0.057
**C12 – H13**	σ	1.97135	**C16 – N18**	σ ∗	3.28	0.83	0.047
**C21 – H24**	σ	1.98683	**C16 – N18**	σ ∗	4.00	0.84	0.052
**O14**	LP	1.95852	**C1 – C12**	σ ∗	8.39	0.70	0.069
**O14**	LP		**C12 – H13**	σ ∗	2.30	0.68	0.036
**N18**	LP	1.95852	**O14 – H15**	σ ∗	4.10	0.77	0.050
**ˮ**	LP		**C16 – H17**	σ ∗	6.63	0.73	0.062
**C1 – C2**	π	1.97397	**C2**	π∗	3.47	0.43	0.084
**ˮ**	π		**C12 – C16**	π∗	1.39	0.31	0.043
**C3 – C4**	π	1.98015	**C3**	π∗	2.91	0.43	0.078
**ˮ**	π		**C4**	π∗	2.87	0.42	0.077
**C5 – C6**	π	1.97895	**C5**	π∗	2.86	0.47	0.081
**ˮ**	π		**C6**	π∗	2.07	0.46	0.068

The non bonded electrons in bonded system would be making electronic transitions among the molecular entities which are used to determine the exchange of required chemical potential for causing drug activity. The first energy transition taking place between C1 – C2 to C3 – C4 & C5 – C6 using absorbed energy of 21.02 and 20.14 kcal/mol. in π- π∗ interacting system. This was the maximum amount of energy in the ring was found to be exchanged with occupation energy of 1.97 kcal/mol this was over leaded the transitions from C1–C2 to C1–C6 and C2–C3 by acquiring energy of 3.67 and 3.07 kcal/mol there were number of transitional electronic exchange taking place around the ring by taking energy from 3.19 to 4.89 kcal/mol another transitional motion was observed between C3–C4 and C1–C2 & C5–C6 with the difference of energy of 19.31 and 20.84 kcal/mol in π- π∗ interacting system.

In reverse oscillation, the energy of 20.71 and 19.39 kcal/mol were exchanged from C5–C6 to C1–C2 and C3–C4 which was represented by π-π∗ lobe interacting arrangement. This also was occurred in ring itself and it was appeared to be oscillated in the core hexagonal ring which was sustained in the ring. The unidirectional transitions were found from C6–H11 to C1–C2 and C4–C5 with the energy gap of 4.67 and 3.71 kcal/mol and it was represented by σ - σ ∗ mono orbital complex. Another allied non bonded transitions observed from C12–H13 and C21–H24 to C16–N18 by absorbing energy of 3.28 and 4.0 kcal/mol these observed transitions were appeared around the ring and allied non bonded electrons of the bonded system. The transitions occurred from lone pair O14 to C1–C12 of the chain by consuming energy of 8.39 kcal/mol in LP- σ ∗ interaction species. The lone pair was found to be activated from induced transitions between O14 and N18 to C12–H13 and O14–H15 and for that, 2.30 and 4.10 kcal/mol amount of energy was utilized in the chain. Another chain transition was taking place between N18 to C16–H17 species by taking energy of 6.63 kcal/mol in non bonded system, the exchange of energy was observed to be within the ring or within the chain and feeble amount of energy only was observed between ring and chain and vice versa. These are the main reason for achieving required amount of drug potential to induce antifungal and additional drug activity.

### 4.12 VCD formulation analysis

**Figure 12:**
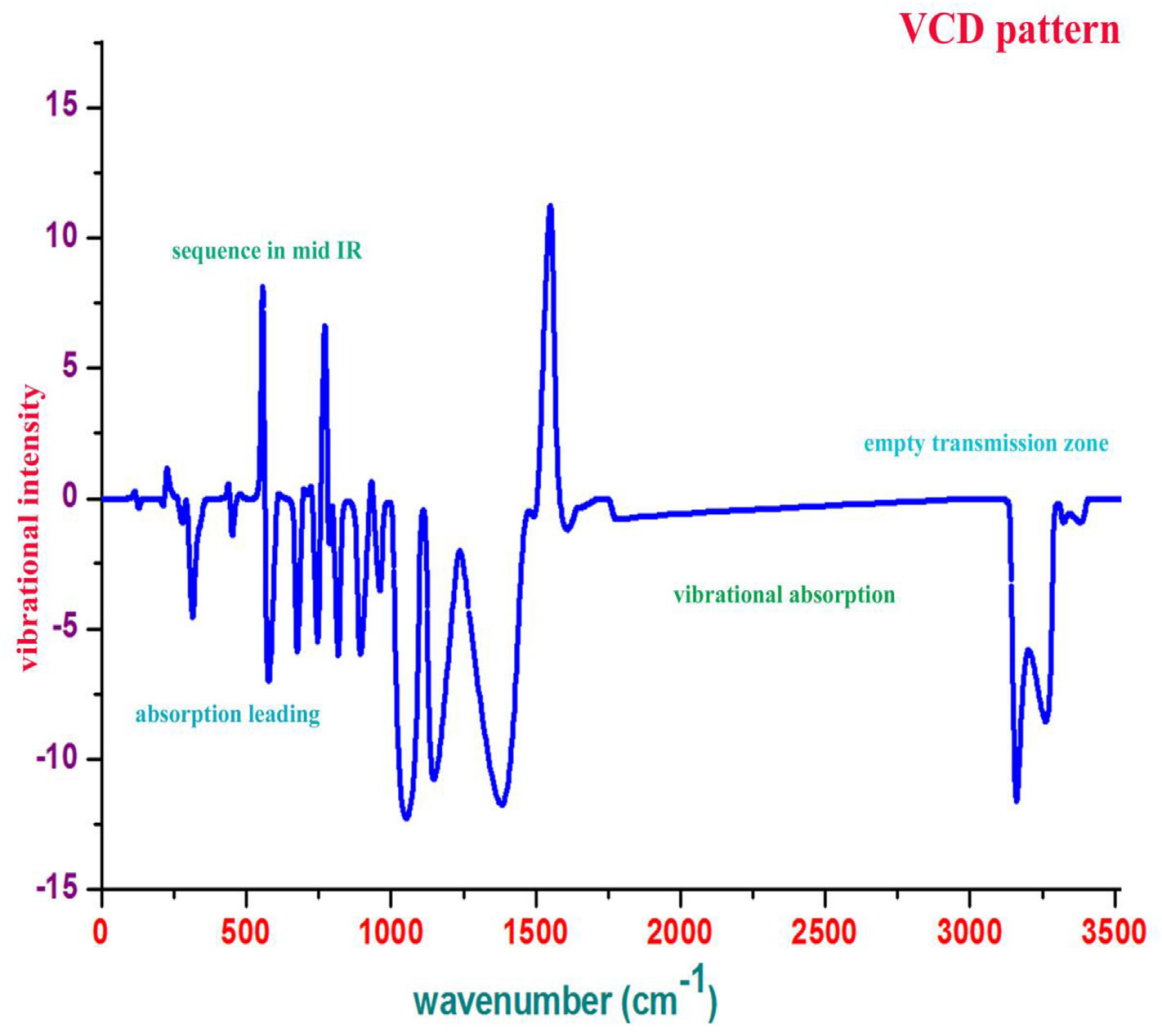
VCD spectra of 2-Amino-1-phenyl-1-propanol.

The vibrational dichroism arrangement for the organic compound represents the vibrational IR absorption and Raman transmission sequence of spectral pattern which directly used to characterize the molecule in terms of chiral character for exposing quantity of toxicity [36]. The absorption and transmission sequence vibrational pattern are differed in various vibrational wavenumber regions which is with respect to the atomic arrangement in molecular symmetry. For the present molecular system, the VCD was shown in Figure 12 in which different vibrational sequence between absorption and transmission was clearly displayed.

According to the chirality rule, the present case is simple and it may be acted as base molecule for advanced drug complex. For satisfying symmetrical enantiomer arrangement, the molecule should have vibrational sequence in all wavenumbers. Accordingly, here, in the region 0-500cm^−1^ and 450-1000 cm^−1^, the symmetrical vibrational sequence was observed. In the case of 1000–1500 cm^−1^ and 3000-3400 cm^−1^, the transmission sequence was found to be missing which showed the asymmetrical vibrational sequence and non enantiomer characteristics of the molecule. The non symmetrical sequence to be solved for reducing toxicity by adding suitable ligand groups in order to form active drug source. It can be achieved some injection of pair of CH_2_ species to the chain or Cl and NH_2_ in ring itself.

## 5 Conclusion

The present organic drug; 2-Amino-1-phenyl-1-propanol was thoroughly analyzed in order to portray structure activity, vibrational character, physical, chemical properties and biological affinity. For that, FT-IR, FT-Raman, UV-Visible and NMR spectral tools have been used along with theoretical tools. The structure viability to describe the drug activity was illustrated by molecular geometry analysis. The charge accumulation over different entities of molecules was exhibited by which the attacks of electrophilic and nucleophilic depleted regions were recognized where the chemical potential stored on molecular entities was validated. The vibrational assignment of different assigned bond length was elucidated by observing the vibrational region from which the involvement of intra-nuclear bonds for organizing the drug activity. The biological parameters have been calculated using different biological tools and from which the Lipinski five rule was satisfied to perceive the biological activity within the molecule. The customized chemical reaction path mechanism to depict the chemical mechanism linked with reactivity for the molecule was monitored by the observation of chemical voids on the core and allied carbons. The driven chemical potential for inducing stabilized drug property and control mechanism inside the frontier molecular moiety-interactive system was elucidated with the help of lobe degenerative sketch. The toxicity level of the compound was described and the controlling mechanism for sustainability of limited toxicity was suggested.

## Declarations

### Author contribution statement

A. Abbas Manthiri: Performed the experiments, Wrote the paper.

S. Ramalingam: Conceived and designed the experiments, Wrote the paper.

Gene George: Analyzed and interpreted the data.

R. Aarthi: Contributed reagents, materials, analysis tools or data.

### Funding statement

This research did not receive any specific grant from funding agencies in the public, commercial, or not-for-profit sectors.

### Data availability statement

Data will be made available on request.

### Declaration of interests statement

The authors declare no conflict of interest.

### Additional information

No additional information is available for this paper.
